# COVID-19 Induced Postural Orthostatic Tachycardia Syndrome (POTS): A Review

**DOI:** 10.7759/cureus.36955

**Published:** 2023-03-31

**Authors:** Deobrat Mallick, Lokesh Goyal, Prabal Chourasia, Miana R Zapata, Kanica Yashi, Salim Surani

**Affiliations:** 1 Internal Medicine, Christus Spohn Hospital, Corpus Christi, USA; 2 Hospital Medicine, Christus Spohn Hospital, Corpus Christi, USA; 3 Hospital Medicine, Mary Washington Hospital, Fredericksburg, USA; 4 Internal Medicine, University of the Incarnate Word School of Osteopathic Medicine, Corpus Christi, USA; 5 Internal Medicine, Bassett Health Care, Cooperstown, USA; 6 Anesthesiology, Mayo Clinic, Rochester, USA; 7 Medicine, Texas A&M University, College Station, USA; 8 Medicine, University of North Texas, Dallas, USA; 9 Internal Medicine, Pulmonary Associates, Corpus Christi, USA; 10 Clinical Medicine, University of Houston, Houston, USA

**Keywords:** postural orthostatic tachycardia syndrome, covid-19 vaccine, pots, sars-cov-2, covid-19

## Abstract

POTS (Postural Orthostatic Tachycardia Syndrome) is a multisystem disorder characterized by the abnormal autonomic response to an upright posture, causing orthostatic intolerance and excessive tachycardia without hypotension. Recent reports suggest that a significant percentage of COVID-19 survivors develop POTS within 6 to 8 months of infection. Prominent symptoms of POTS include fatigue, orthostatic intolerance, tachycardia, and cognitive impairment. The exact mechanisms of post-COVID-19 POTS are unclear. Still, different hypotheses have been given, including autoantibody production against autonomic nerve fibers, direct toxic effects of SARS-CoV-2, or sympathetic nervous system stimulation secondary to infection. Physicians should have a high suspicion of POTS in COVID-19 survival when presented with symptoms of autonomic dysfunction and should conduct diagnostic tests like the Tilt table and others to confirm it. The management of COVID-19-related POTS requires a comprehensive approach. Most patients respond to initial non-pharmacological options, but when the symptoms become more severe and they do not respond to the non-pharmacological approach, pharmacological options are considered. We have limited understanding and knowledge of post-COVID-19 POTS, and further research is warranted to improve our understanding and formulate a better management plan.

## Introduction and background

The COVID-19 pandemic has affected millions since December 2019 [1]. Long-term effects with adverse outcomes of COVID-19 are increasingly being recognized [2-4]. These long-term effects can be seen in patients with mild to severe disease [4-6]. Though the SARS-CoV-2 virus primarily affects the respiratory system, it can affect multiple organ systems in the body, including the autonomic system and nervous system [7]. The harmful effects of COVID-19 on autonomic nervous systems can continue to persist even after the resolution of an acute COVID-19 infection and can be very debilitating [7]. Effects on the autonomic and nervous systems can adversely affect health and quality of life in patients with a history of COVID-19 infection. One of these manifestations that is increasingly getting recognized is POTS syndrome [8-11].

POTS (Postural Orthostatic Tachycardia Syndrome) refers to the development of orthostatic symptoms associated with an increase in heart rate greater than or equal to 30 (from normal resting heart rate) but not associated with orthostatic hypotension [12]. The mechanisms proposed to explain POTS include autonomic neuropathy, increased sympathetic tone, a hypovolemic state with an altered renin-angiotensin-aldosterone system, and autoimmunity [13-17]. With the reporting of an increasing number of cases, POTS is gaining recognition in patients with COVID-19 in the post-infectious stage [8]. SARS-CoV-2 can access multiple organ systems through the ACE-2 receptor [18]. Its pathophysiology is postulated to include virus- or immune-mediated damage to the autonomic nervous system.

Autonomic dysfunction, of which POTS is an important subset, has been noted in more than half the patients with COVID-19 as post-acute sequelae in some studies [19]. Understanding these critical sequelae of COVID-19 for early diagnosis and effective management is essential. Given the limited literature to date, we aim to comprehensively review current literature and treatment strategies to synthesize the available evidence and enhance understanding of this critical and debilitating condition in patients with POTS after COVID-19 infection.

## Review

Methodology

The literature search was performed using PubMed, Science Direct, and Google Scholar search engines. The search syntax included the following terms: “Covid-19” and “POTS,” “Postural Orthostatic Tachycardia Syndrome”, “symptoms”, “prevention”, and “treatment”. Three physician authors reviewed the literature, and the information is summarized in this article for simplicity of comprehension.

Pathophysiology

Normal Orthostatic Response

When you stand against gravity, approximately 500 to 800 ml of blood is displaced to the abdomen and lower extremities [20,21]. In a normal response, our autonomic nervous system (sympathetic and parasympathetic) gets activated to compensate for and maintain cardiac output by increasing heart rate and peripheral vasoconstriction. At the same time, muscles of the abdomen and lower extremity also contract rhythmically and squeeze capacitance vessels to help the blood return to the heart.

Mechanism of POTS: There are multiple mechanisms and pathophysiologic conditions that are responsible for POTS. The patient can have one or more of these conditions:

*Hypovolemia and deconditioning:* Decreased intravascular volume has been seen in the majority of POTS patients, which, in turn, reduces cardiac venous return and initiates reflex tachycardia [16,17]. Patients with severe deconditioning further exacerbated hypovolemic symptoms.

*Autoimmunity:* Many patients, after a viral illness, develop POTS symptoms, which are suspected to be due to an autoimmune response. Few of the patients were detected to have autoantibodies to adrenergic, acetylcholine, and angiotensin II receptors [12,15]. These antibodies cause peripheral vasoconstriction to be less effective and eventually cause reflex tachycardia. It has been observed that many patients with COVID-19 infection develop POTS as a delayed manifestation [9-11,19,22]. Some of these patients have never experienced any symptoms of POTS before.

*Neuropathy:* It is estimated that up to half of the POTS cases have some form of small-fiber neuropathy, resulting in dysautonomia. Up to 43% to 63% of POTS patients with GI symptoms have abnormal sudomotor testing [23-27]. Similarly, POTS patients have impaired adrenergic nervous function [27].

*Neuroendocrine dysfunction:* As compared to healthy people, POTS patients have evidence of higher levels of catecholamines [28] and angiotensin II [29] and lower levels of plasma renin and aldosterone [14,17,23] at baseline. There is also evidence that with standing POTS, patients have an increase in norepinephrine levels.

Discussion

Post-COVID Conditions (PCC) encompass a variety of sequelae or health conditions that last four weeks or more after a SARS-CoV-2 infection [30]. Although it can affect many different body systems, it can ultimately cause autonomic dysfunction, which can lead to postural orthostatic tachycardia syndrome (POTS) [30]. 

Possible mechanisms of post-COVID-19 POTS

The exact mechanism causing post-COVID-19 POTS is still not clear, but several mechanisms have been suggested.

*Autoimmunity* is one of the most likely mechanisms by which a coronavirus triggers the production of autoantibodies against autonomic nerve fiber, adrenergic, acetylcholine, and angiotensin II receptors [22,31,32]. There is another mechanism, which is the direct toxic action of the COVID-19 virus, resulting in tissue injuries [33]. A spike protein of the COVID-19 virus attached to ACE2 receptors enters the cell and causes multisystem damage, resulting in dysregulation of the RAAS system [33]. The spike proteins of the COVID-19 virus also exhibit neurotoxic effects and can produce POTS symptoms [34,35].

**Figure 1:**
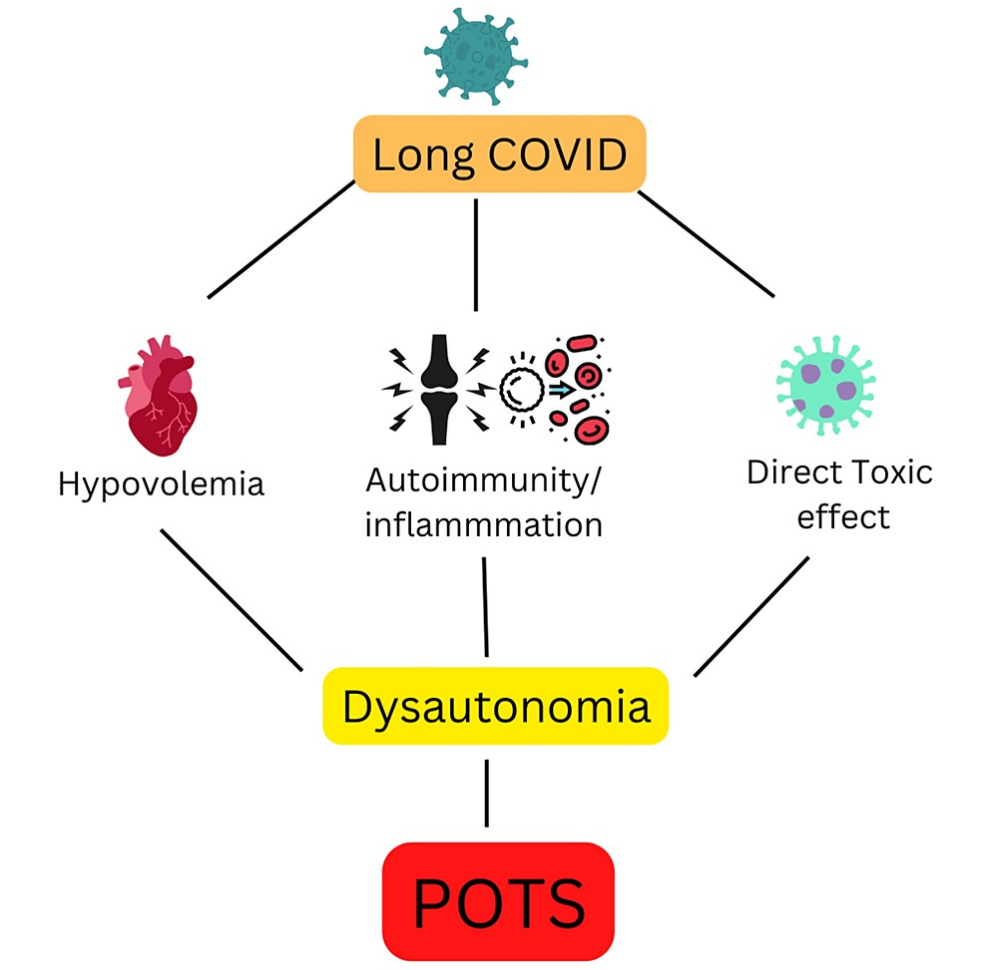
Possible mechanisms of COVID-induced POTS Author's own creation

The neuroinvasive capabilities of the COVID-19 virus are well known [36,37]. The COVID-19 virus can directly invade the CNS and ANS via the olfactory nerve and the ACE2 receptor in the brainstem or indirectly through the enteric nervous system via GI tract infection [38-40]. The brainstem has a major role in the regulation of the cardiovascular system, autonomic nervous system, and neurotransmitter systems [41]. Damage to the brainstem results in dysregulation of these systems, resulting in POTS symptoms. Furthermore, COVID-19 infection can cause hypovolemia, decreased cardiac output, and sympathetic nervous system activation secondary to the fever, night sweats, nausea, and vomiting was usually seen in patients with COVID-19 infection [8,42]. Cytokine hyperactivation, which is seen in patients with COVID infection, also results in sympathetic nervous system stimulation, resulting in POTS symptoms [43]. Figure 1 shows the possible mechanisms of COVID-induced POTS.

Epidemiology

Patients diagnosed with POTS syndrome are usually younger (age 18-45) and female. The female-to-male ratio of patients affected by POTS syndrome is 6:1 [44]. A recent large cross-sectional study showed that more than 90% of the patients affected were white females of childbearing age [44].

Clinical presentation

The clinical presentation of POTS consists of two types, orthostatic and non-orthostatic symptoms: 

*Orthostatic: *These symptoms resulted from decreased cerebral perfusion and increased sympathetic stimulation that occurs when the patient stands and is relieved when they lie down [12]. Common orthostatic symptoms include feeling dizzy, heart fluttering or palpitations, fainting, changes in sweating pattern (either too much or too little), trouble breathing, and chest pain [12]. These manifestations can worsen with physical activity, eating meals, bathing, hot temperatures, and during one’s menstrual cycle [12].

*Non-orthostatic: *These symptoms are typically not associated with changes in cerebral perfusion or sympathetic activation and are independent of individual positioning [12,45]. Some symptoms include anxiety, severe headaches, and changes in sleeping habits [12,45]. Several other body systems can be affected, resulting in a wide variety of symptoms, as mentioned in detail below:

*Psychological dysfunction:* Anxiety is a common symptom seen in POTS and other autonomic disorders [46]. It has been suggested that anxiety develops due to either excessive awareness and apprehension of physical symptoms related to orthostasis or a conditioned fear response resulting in tachycardia when individuals resume an upright position [46,47]. 

*Fatigue:* While this can occur at rest, fatigue can also be accompanied by orthostatic symptoms in patients with a low tolerance for physical activity [12]. Fatigue can occur in cycles and may last days to weeks before it resolves [12].

*Gastrointestinal (GI) dysfunction*: This includes changes in bowel habits such as constipation or diarrhea, nausea, vomiting, and stomach pain [48]. Although there is not a clear understanding of why GI symptoms occur, management of POTS may improve GI discomfort [48].

*Cognitive dysfunction:* Patients can complain of mental fog and may demonstrate mild to moderate impairment in cognition and depression on a neuropsychological evaluation [49]. This may be a result of catecholamine pathway dysfunction or abnormalities in various brain structures [49].

*Bladder dysfunction:* Overactive bladder symptoms consist of nocturia, frequency, and urgency [50]. This is suggested to be caused by autonomic dysfunction of the lower urinary tract [50]. 

*Dermatologic manifestations:* Common skin changes include hives, Raynaud’s phenomenon, and livedo reticularis [51]. These are a result of sympathetic activation, increased mast cell degranulation, or vasoconstriction of cutaneous blood vessels [51].

Diagnosis

There are three basic criteria for diagnosing patients with possible POTS:

*Symptomatic orthostatic tachycardia:* Patients suspected of having POTS will have a heart rate elevation > 30 beats/minute (but greater than 40 bpm in patients < 20 years old) about their baseline when standing without orthostatic hypotension [45,52]. In orthostatic hypotension, we see a reduction of standing systolic or diastolic blood pressure by 20 mmHg or 10 mmHg, respectively. However, in POTS, the blood pressure remains stable or increases when standing. The only change noticed is in the patient's heart rate and symptoms of orthostatic intolerance.

A normal, healthy person can have a transient increase in heart rate for 20 seconds without any symptoms. Whereas in patients with POTS syndrome, a heart rate typically increases between 30 and 60 seconds and continues to rise during standing [13].

During the evaluation of POTS, physicians should also consider the patient's current medications, which can affect the patient's heart rate, for example, beta-blockers, which may suppress the heart rate, and therefore, patients can have a false diagnosis of POTS. Similarly, other medications like anticholinergics, adrenergic stimulants, etc., should also be taken into account as they can falsely increase the patient's heart rate, leading to a false positive POTS diagnosis [53].

*Autonomic testing: *Patients who are suspected of having POTS should undergo autonomic testing. This test is considered more sensitive and specific when compared to a random heart rate when standing during bedside evaluation. The autonomic testing consists of 2 types: Tilt table testing and sudomotor testing. Out of these two, the tilt table is considered to be the best test and easiest to perform.

*Tilt table testing*: This test provides objective evidence of orthostatic intolerance and helps exclude orthostatic hypotension. In this test, the patient is secured on the table and lying flat. The table is then slowly raised to an upright position. The test measures the patient’s heart rate, blood pressure, blood oxygen levels, and exhaled carbon dioxide levels. The patient is diagnosed with POTS if he/she has an abnormally elevated heart rate (i.e., > 30 beats per minute when compared to resting heart rate), worsening of symptoms in an upright position, and is negative for orthostatic hypotension.

*Sudomotor testing*: This test measures the autonomic nerves, which are responsible for sweat regulation.

*Laboratory values:* Orthostatic intolerance symptoms are not specific to patients with POTS. Patients suspected of having POTS should undergo laboratory testing to rule out other causes of orthostatic intolerance symptoms. These tests include [12,28]: Complete blood count, comprehensive metabolic panel, thyroid function tests: to rule out hypo- and hyperthyroidism, electrocardiography (EKG/ECG): to rule out underlying heart conditions, morning cortisol levels, transthoracic echocardiogram: to rule out heart failure, plasma catecholamines, urine sodium, and/or 24-hour urine sodium.

Differential diagnosis

Due to the wide range of symptoms, other clinical conditions could present similarly and need to be ruled out:

*Orthostatic hypotension*: This is characterized by changes in blood pressure, such as >20 mmHg systolic or >10 mmHg diastolic [54]. The heart rate will compensate for positional changes differently depending on whether the cause is neurogenic or non-neurogenic [54]. Although both share orthostatic symptoms, orthostatic hypotension is more characteristic of changes in blood pressure, whereas POTS is reflective of changes in heart rate.

*Hypovolemia*: Patients with dehydration usually present with an elevated heart rate, which could give a false-positive diagnosis of POTS. Therefore, we should avoid diagnosing POTS in patients with an acute or chronic diagnosis of dehydration, such as polyuria, vomiting, dehydration, etc. Dehydrated patients will also typically present with orthostatic hypotension due to intravascular volume depletion, resulting in tachycardia as a reflex to maintain the same cardiac output. Orthostatic hypotension is already an exclusion criterion for the diagnosis of POTS. Some drug classes, such as loop diuretics, thiazides, stool softeners, etc., when taken in excessive amounts than the recommended dose, can result in hypovolemia and therefore cause reflex tachycardia, which also leads to hypotension [55]. 

*Medication-induced tachycardia*: Several medications can induce tachycardia and lead to the misdiagnosis of POTS. Stimulants such as methamphetamine, cocaine, and caffeine can increase heart rate through catecholamine mechanisms and by inhibiting phosphodiesterase, respectively [56]. Commonly used bronchodilators such as albuterol can increase heart rate through the mechanism of activating beta-adrenergic receptors [56]. Antidepressants, including fluoxetine and escitalopram, can also cause tachycardia because of sodium and calcium inhibition [56]. Lastly, several cardiac medications like digoxin and milrinone can increase vasodilation and heart contractility via Na+/K+ ATPase inhibition and phosphodiesterase inhibition, respectively [56]. 

*Inappropriate sinus tachycardia (IST)*: This condition is defined as having greater than 100 bpm at rest and having over 90 bpm on average over 24 hours [57]. This contrasts with POTS due to the tachycardia being present at baseline and not just after positional changes, such as standing [57]. 

*Reflex syncope*: Patients that experience vasovagal syncope can display orthostatic symptoms such as sweating, nausea, or dizziness, but it typically occurs after standing for long periods [58].

*Psychological conditions*: Increased heart rate and feelings of palpitations can occur in psychiatric conditions such as anxiety but also overlap with the profile of symptoms seen in POTS [49]. It is still unclear if this is due to a high prevalence of anxiety as a coexisting condition in patients with POTS or if there is just an overlap of symptoms between the two conditions [49]. 

COVID-19 vaccine-induced POTS

The best method, which is considered the safest and most effective against COVID-19 infection, is vaccination. Multiple studies have shown that COVID-19 vaccinations have reduced hospitalizations, complications, and death in patients infected with the SARS-CoV-2 virus [59]. However, there have been a few case reports published in peer-reviewed journals that show patients affected with POTS after receiving mRNA COVID-19 vaccination. The exact mechanism of action is not known at this time, but an autoimmune response to vaccination is one of the possible mechanisms. Even though few case reports exist about POTS due to COVID-19 vaccination, the evidence is not strong enough currently [60,61]. A recent cohort study performed on 284,592 individuals showed that the incidence of POTS is high 90 days after the COVID-19 vaccine when compared to 90 days before the vaccine. An individual with SARS-CoV-2 infection has five times the chance of being affected by POTS when compared to vaccinated individuals who get infected with SARS-CoV-2 [62].

Management

The current guidelines for the management of post-COVID-19 POTS and non-COVID-19-related POTS are the same. The management of POTS requires a comprehensive approach. Initially, nonpharmacological options should be tried, but when symptoms are severe, pharmacological agents may be needed [7].

Nonpharmacological management

*Intravascular volume expansion by salt and water intake:* It is suggested that patients with POTS should be encouraged to have a daily oral fluid intake of up to 3 L/day and sodium chloride of up to 8 to 12 g [63-65].

For patients who cannot tolerate oral fluid, an IV bolus of 1 to 2 L of normal saline can be used as a short-term strategy for up to two days [66-68].

*Physical exercise:* Studies have demonstrated improvement of POTS symptoms with aerobic exercise programs with gradual increments as tolerated [12,63,69,70]. Patients can also exercise in a semi-recumbent position if they cannot tolerate an upright position.

*Lifestyle modification:* Different strategies can be followed, like avoiding prolonged bed rest and daytime sleeping and taking measures to improve sleep quality [71]. Cognitive behavioral therapy for any psychiatric symptoms or functional disability [72] and also increase physical activity of the lower extremity to improve venous return.

*Compressive garments:* In more severe cases not responding to initial nonpharmacological treatment, compression garments can be tried before starting on medication. We can use graded compression stockings at 30 to 40 mmHg, or another option are abdominal binders at 10 mmHg. Compressive garments significantly reduce venous pooling and improve POTS symptoms [73]. An abdominal binder is more beneficial compared to leg stockings as compression of the abdomen causes the shifting of a significant amount of pooled venous blood from high capacitance splanchnic veins [74,75].

Pharmacological management

The choice of medication depends on which symptom or disorder we are targeting. 

For patients with orthostatic tachycardia, beta-blockers will be the drug of choice, but for patients with sudomotor or vasomotor symptoms, vasopressin therapy will be more effective [64].

*Beta-blockers:* Both beta-1 selective and nonselective beta-blockers have been used in POTS management. Beta-blockers help by reducing tachycardia and the hyperadrenergic state and improving exercise intolerance and orthostatic intolerance. Commonly used agents are Metoprolol-0.25 to 0.5 mg/kg twice daily and Propranolol 20 mg daily [76,77].

*Ivabradine:* It blocks the channel responsible for the cardiac pacemaker current, thereby reducing heart rate. Usually given the dose of 2.5 to 20 mg/day in two divided doses titrated to achieve resting supine heart rate between 50/min to 70/min. It improves the symptoms of orthostatic tachycardia in POTS patients [78-82].

*Fludrocortisone:* It works by temporarily expanding intravascular volume and increasing peripheral vascular resistance. The usual dose is 0.05 to 0.2 mg daily, thereby improving orthostatic symptoms [83,84].

*Midodrine:* (Alpha agonist) works as a vasopressor agent and reduces peripheral blood pooling and thereby improving orthostatic intolerance. The usual dose is 2.5 to 10 mg three times a day during the daytime when the patient is awake and in an upright position [85,86].

*Pyridostigmine:* It’s a reversible acetylcholinesterase inhibitor usually given in a dose of 30 to 60 mg three times per day. It improves orthostatic heart rate and clinical symptoms of orthostatic intolerance in POTS patients [87].

*Droxidopa:* Acts as a prodrug to the neurotransmitter norepinephrine and causes peripheral vasoconstriction. The usual dose is 100 milligrams three times a day, but you may titrate up to 600 mg thrice a day if blood pressure tolerates. It improves hypovolemia and orthostatic intolerance [88].

*Methylphenidate:* Long-acting alpha agonist and releases catecholamines in the synapses. It improves the symptoms of presyncope, orthostatic palpitation, and fatigue. Usually given, a dose of 10 mg twice daily may increase up to 30 mg twice daily [89].

*SSRIs:* It improves the vasoconstriction reflex by stimulating nerve communication. This eventually reduces venous pooling and improves orthostatic intolerance [90,91].

Treatments no longer indicated

Previously several invasive and noninvasive procedures and intervention been used for the treatment of POTS but has no longer been used due to high risk and lack of efficacy like Vagal nerve stimulation, radiofrequency sinus node modification, and thoracic venous system endovascular intervention [65,92,93]. 

Special circumstances in POTS patient

Effect of Pregnancy on POTS Patients

The gravid uterus, especially in the third trimester, can compress against the inferior vena cava, which in return decreases venous return to the heart and cardiac output, resulting in further worsening of orthostatic symptoms in pregnant POTS patients. In pregnancy, nonpharmacological therapy is most appropriate. Discussion with an obstetrician is very important before starting any pharmacological treatment. If there is no evidence of hypertension, preeclampsia, or eclampsia, we should continue with salt supplementation during pregnancy [94].

*POTS patient undergoing surgery*: There is no clear guideline for perioperative management; however, there are some recommendations for POTS patients undergoing surgery, such as patients undergoing surgery should receive IV fluid bolus when they have fluid oral restriction per preoperative protocol guidelines. Also, due to the high incidence of hypokalemia in patients on fludrocortisone, the patient's potassium levels should be monitored [95]. 

*Anesthesia*: More study is needed about the anesthesia effect on POTS patients. We do not have sufficient evidence about the superiority of one anesthetic agent over another [95]. 

## Conclusions

Now that we have moved past the peak of the COVID-19 pandemic, we are now seeing several post-COVID conditions, including Postural Orthostatic Tachycardia Syndrome (POTS). This comprehensive review is beneficial for providing general information on clinical symptoms, diagnosis, and management of POTS, as well as increasing awareness of this prevalent post-COVID condition.

POTS may be challenging to identify due to the mixed clinical picture of symptoms that result from orthostatic and non-orthostatic mechanisms. Although our understanding is not entirely clear, some postulated mechanisms consist of autoantibody production against autonomic nerve fibers, direct toxic effects of SARS-COV-2, or sympathetic nervous system stimulation secondary to infection. When evaluating for POTS, it is important to review current medications and obtain labs, including cortisol and normetanephrine levels, to rule out secondary causes of orthostatic intolerance. Management consists of nonpharmacologic approaches and medications depending on predominant symptom characteristics. Overall, increased clinical awareness will lead to earlier diagnosis and better management of POTS after COVID-19 infection.
